# A Deep Learning Model to Predict the Response to Neoadjuvant Chemoradiotherapy by the Pretreatment Apparent Diffusion Coefficient Images of Locally Advanced Rectal Cancer

**DOI:** 10.3389/fonc.2020.574337

**Published:** 2020-10-29

**Authors:** Hai-Tao Zhu, Xiao-Yan Zhang, Yan-Jie Shi, Xiao-Ting Li, Ying-Shi Sun

**Affiliations:** Department of Radiology, Key Laboratory of Carcinogenesis and Translational Research (Ministry of Education/Beijing), Peking University Cancer Hospital & Institute, Beijing, China

**Keywords:** rectal cancer, magnetic resonance imaging, apparent diffusion coefficient, deep learning, good responder

## Abstract

**Background and Purpose:**

Pretreatment prediction of the response to neoadjuvant chemoradiotherapy (NCRT) helps to determine the subsequent plans for the patients with locally advanced rectal cancer (LARC). If the good responders (GR) and non-good responders (non-GR) can be accurately predicted, they can choose to intensify the neoadjuvant chemoradiotherapy to decrease the risk of tumor progression during NCRT and increase the chance of organ preservation. Compared with radiomics methods, deep learning (DL) may adaptively extract features from the images without the need of feature definition. However, DL suffers from limited training samples and signal discrepancy among different scanners. This study aims to construct a DL model to predict GRs by training apparent diffusion coefficient (ADC) images from different scanners.

**Methods:**

The study retrospectively recruited 700 participants, chronologically divided into a training group (n = 500) and a test group (n = 200). Deep convolutional neural networks were constructed to classify GRs and non-GRs. The networks were designed with a max-pooling layer parallelized by a center-cropping layer to extract features from both the macro and micro scale. ADC images and T2-weighted images were collected at 1.5 Tesla and 3.0 Tesla. The networks were trained by the image patches delineated by radiologists in ADC images and T2-weighted images, respectively. Pathological results were used as the ground truth. The deep learning models were evaluated on the test group and compared with the prediction by mean ADC value.

**Results:**

Area under curve (AUC) of receiver operating characteristic (ROC) is 0.851 (95% CI: 0.789–0.914) for DL model with ADC images (DL_ADC), significantly larger (P = 0.018, Z = 2.367) than that of mean ADC with AUC = 0.723 (95% CI: 0.637–0.809). The sensitivity, specificity, positive predictive value (PPV) and negative predictive value (NPV) of DL_ADC model are 94.3%, 68.3%, 87.4% and 83.7%, respectively. The DL model with T2-weighted images (DL_T2) produces an AUC of 0.721 (95% CI: 0.640–0.802), significantly (P = 0.000, Z = 3.554) lower than that of DL_ADC model.

**Conclusion:**

Deep learning model reveals the potential of pretreatment apparent diffusion coefficient images for the prediction of good responders to neoadjuvant chemoradiotherapy.

## Introduction

Locally advanced rectal cancer (LARC) is defined as rectal cancer with clinical tumor stage 3-4 (cT3-cT4, tumor invades through the muscularis propria) or positive clinical nodal stage (cN+, malignant lymph nodes are detected). Preoperative neoadjuvant chemoradiotherapy (NCRT) is the standard treatment procedure for LARC patients (1–3). Some good responders (GR) may achieve pathological tumoral stage 0-1 (ypT0-1, muscularis propria is not invaded) and negative pathological nodal stage (ypN0, no malignant lymph nodes are found) after NCRT. These GRs may avoid total mesorectal excision (TME) surgery by using “wait and see” strategy or local excision to preserve organs and improve the life quality (4, 5). Several methods have been proposed to predict the pathological complete response (6–10), pathological good responses (11–13), lymph node metastasis (14, 15) of LARC by using two sets of MRI data, one before the initiation of NCRT and another during or after NCRT. In addition to assessing response after or during NCRT, it is also beneficial to predict GRs before the start of NCRT. If GRs and non-good responders (non-GR) could be classified before the initiation of NCRT, individualized treatment could be implemented to each classification to maximize their benefit from NCRT.

Several radiomics methods have been proposed to discriminate GRs and non-GRs based on MRI data before the initiation of NCRT. (16–21). In these works, classification models were constructed by handcrafted features such as shape, gray histogram and texture. But these generalized features are not specially designed for rectal MRI data. The development of deep learning (DL) makes it possible to adaptively extract features without the need of predefinition. A study has shown that VGG19 networks pre-trained by ImageNet dataset (AUC = 0.73) yield significantly larger AUC than handcrafted features (AUC = 0.64) (22). However, it is still a challenging problem to train DL networks by rectal MRI data because DL requires much more data than conventional machine learning. If data from multiple scanners are used, the signal variation in magnetic field or venders cannot be ignored. A study based on two 1.5 Tesla scanners has found that 75% of features are unstable to variations in vendors and image acquisition protocols (21). The variations are larger if images are scanned at different magnetic field strength. If DL is trained by data with considerable discrepancy, the networks may fail to reach the optimal condition.

Compared with T2-weighted images or diffusion-weight images, apparent diffusion coefficient (ADC) is an inherent physical value of the tissues and is independent on the scanning conditions. Therefore, it could be expected that the DL model based on ADC images is insensitive to the difference in scanning conditions. In this study, a DL model was proposed to classify GRs and non-GRs to NCRT by the pretreatment MRI data of rectal cancer. The networks were trained by ADC images of 500 participants from a 1.5 Tesla scanner and a 3.0 Tesla scanner. A chronologically separated test group with 200 participants was used to validate the performance of the model. The same networks trained by T2-weighted images were used for comparison.

## Materials and Methods

### Subjects

**Figure 1:**
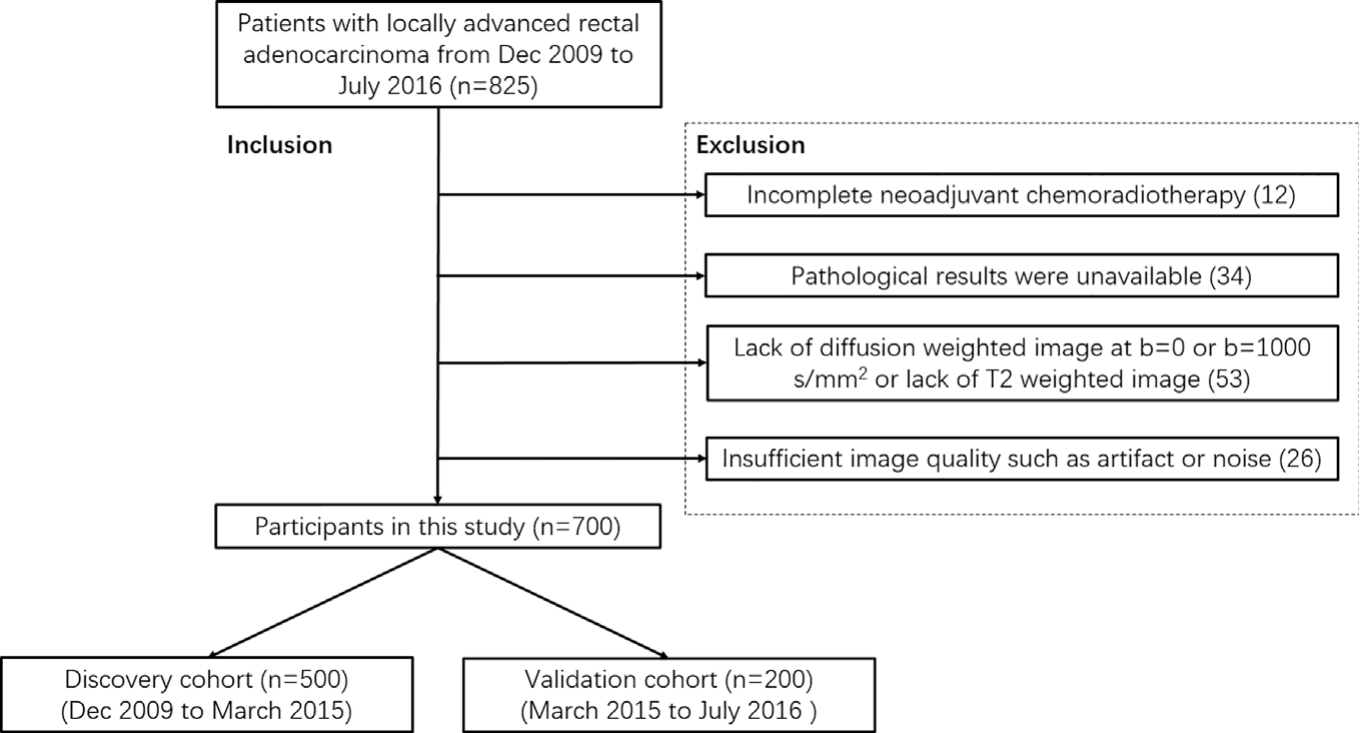
Flowchart of inclusion and exclusion.

This retrospective study enrolled 700 participants with rectal cancer from Dec 2009 to July 2016. The protocol has been approved by the medical ethics committee of Beijing Cancer Hospital. All candidates satisfied the following criteria: (a) proven as locally advanced rectal adenocarcinoma by histopathology and baseline MRI examination (≥cT3 or cN+); (b) scheduled to NCRT in our hospital. Participants were excluded if (a) NCRT was incomplete; (b) pathological results were unavailable; (c) Lack of diffusion-weighted image at b = 0 or b = 1000 s/mm^2^ or lack of T2-weighted image; (d) the quality of images is insufficient for measurement due to artifacts or noise. All participants (n = 700) were divided into a training group (n = 500, from December 2009 to March 2015) and a test group (n = 200, from March 2015 to July 2016) chronically. **Figure 1** is the flowchart of inclusion and exclusion.

### Scanning Parameters

All participants underwent MRI within one week before initiation of NCRT. 221 participants were scanned on a 1.5 Tesla MRI scanner (Optima MR360) and 479 participants were scanned on a 3.0 Tesla MRI scanner (Discovery MR750) using an 8-channel phased array body coil in the supine position. The scanning parameters of diffusion-weighted imaging (DWI) and T2-weighted imaging (T2WI) are summarized in **Table 1**.

**Table 1:** Scanning parameters of T2-weighted imaging (T2WI) and diffusion-weighted imaging (DWI) protocols on 1.5 Tesla and 3.0 Tesla scanners.

	1.5 Tesla		3.0 Tesla	
	DWI	T2WI	DWI	T2WI
**TR (second)**	5.0–6.0	3.6–4.8	2.8	4.8–5.7
**TE (millisecond)**	65–80	100–110	66	100–110
**FOV (mm)**	340	180	340	180
**Echo Train Length**	1	16	1	25
**b value (s/mm^2^)**	0,1000	–	0,1000	–
**Image Size**	256 × 256	256 × 256	256 × 256	512 × 512
**Thickness (mm)**	5.0	3.0	4.0	3.0
**Gap (mm)**	1.0	0	1.0	0.3

### ROI Delineation

**Figure 2:**
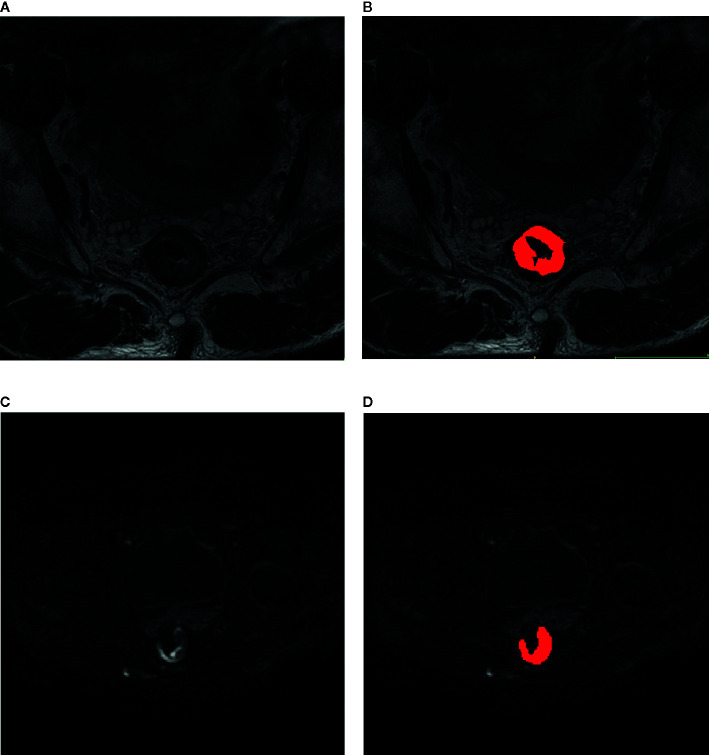
Delineation of region of interest on the images of rectal cancer. **(A)** T2-weighted image; **(B)** T2-weighted image overlaid by the manual delineation. **(C)** diffusion-weighted image with b-value of 1000 sec/mm^2^. **(D)** Diffusion-weighted image with b-value of 1000 sec/mm^2^ overlaid by the manual delineation.

Region of interests (ROI) of rectal tumor were manually delineated on each slice of T2-weighted images and diffusion-weighted images at b-value of 1000 sec/mm^2^ by two experienced radiologists with ITK-SNAP (http://www.itksnap.org/). Tumor shows slightly high signal on T2-weighted images and shows high signal on diffusion-weighted images at b-value of 1000 sec/mm^2^. The region of intestinal lumen was excluded. Images of other protocols were used as reference to exclude false positive signals. Randomly selected 50 subjects were used to train the two radiologists to reach a dice similar coefficient larger than 0.9. An example of ROI delineation was shown in **Figure 2**.

### Neoadjuvant Chemoradiotherapy (NCRT)

All participants received 22-fraction (5 times per week) of 2.3 Gy (gross tumor volume) and 1.9 Gy (clinical target volume) intensity-modulated radiation therapy using a Varian Rapidarc system (Varian Medical Systems). 825 mg/m^2^ capecitabine was orally given twice every day concurrently with radiation therapy. All participants received TME surgery within 8–10 weeks after completion of NCRT.

### Pathological Assessment

After TME, surgically resected specimens were examined and analyzed by two pathologists in consensus. Pathological results were used as ground truth to define GR (ypT0-1 and ypN0) and non-GR (ypT>1 or ypN>0).

### Image Pre-Processing

ADC images were calculated by images at b = 0 and image at b = 1000 sec/mm^2^ with an in-slice 3 × 3 convolutional kernel for smoothing to eliminate the possible misregistration between two images. ROI delineated at b = 1000 sec/mm^2^ was directly used as the ROI of ADC images. T2-weighted images were normalized to the range between 0 and 1 to minimize the difference among the scanners. The smallest cuboid that contain the whole delineation was cropped. 6 mm margins were added to the slice plane. All image patches were reshaped to the size of 64 × 64 × 16 by zero-padding. Data augmentation was performed by rotating the ROI by *N* times with each of 360/*N* degrees. *N* is 100 for GRs, and *N* is 25 for non-GRs according to the ratio of two classes to keep a balance in training.

### Deep Convolutional Neural Network

**Figure 3:**
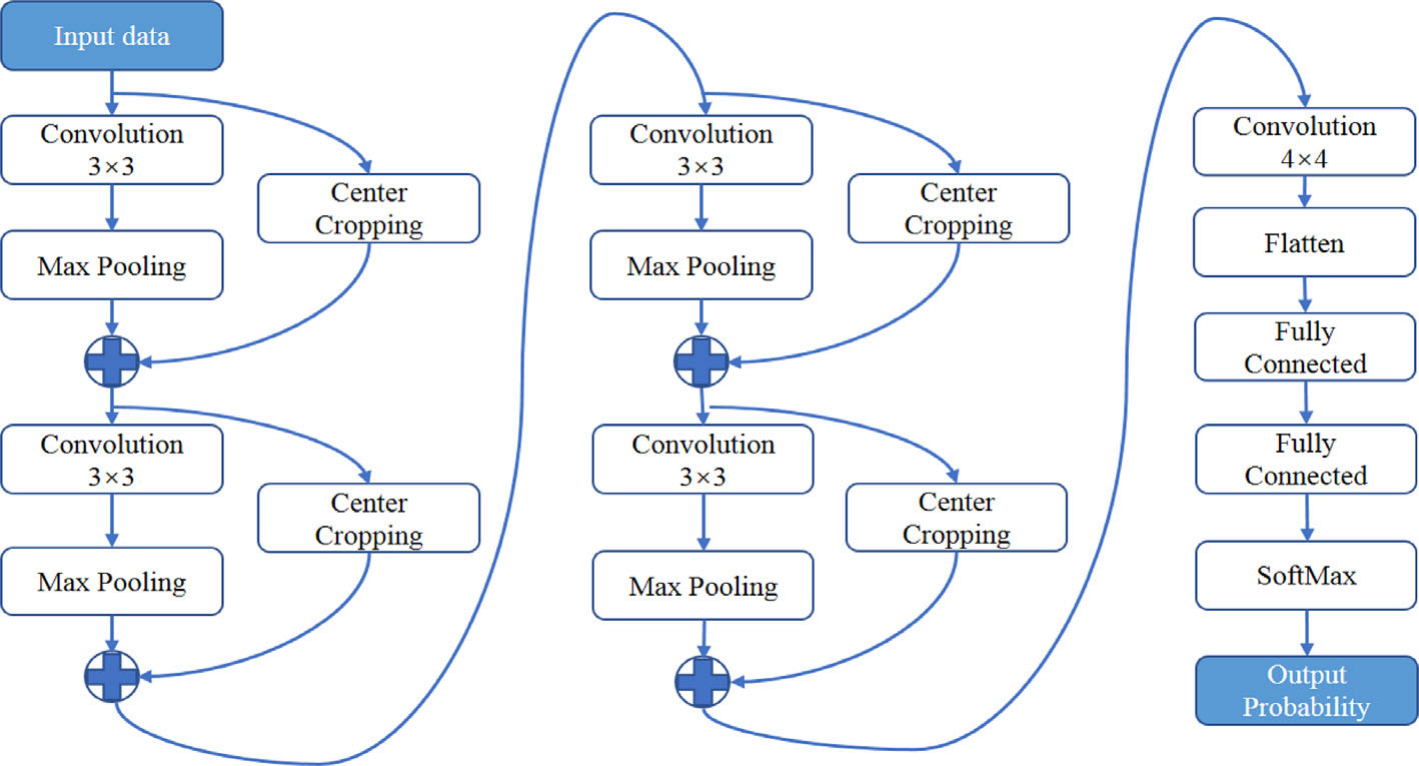
The architecture of neural networks for deep learning. A feature-extract unit is designed with a convolution followed by a max-pooling layer and paralleled by a center-cropping layer. The networks contain four repetitions of the feature-extract units.

Processed ADC patches or T2-weighted patches were inputted into the convolutional neural networks shown in **Figure 3**. A feature-extract unit is designed with a convolution followed by a max-pooling layer and paralleled by a cropping layer. The size of convolution kernel is 3 × 3. The size of max-pooling is 2 × 2, which passes the maximal value of each 2 × 2 voxels into the next layer. The cropping layer extracts the central 1/4 region of the image into next layer and concatenated with the output of max-pooling layer. This structure manages to extract information from both the whole image and the central area. After 4 repetitions of the feature-extract units, dense connected layers and a SoftMax function was used at final to produce a probability between 0 and 1. The network architecture was implemented using Python 3.6 based on Keras 2.1.5 with TensorFlow 1.4.0 as its backend. The network was trained by stochastic gradient descent algorithm with the adaptive moment estimation and a binary cross-entropy loss function. 64G memory and a graphic processing unit (GPU: NVIDIA TITAN XP 12G) was used. It took averagely 10 hours to train the networks.

The training group is randomly divided into five subgroups for 5-fold cross-validation to determine the optimal hyperparameters for training. For each fold, four subgroups (400 samples) were used for training and the other subgroup (100 samples) was used for validation. The hyperparameters (learning rate, decay rate, batch size, and number of epochs) corresponding to the largest mean accuracy were used for subsequent construction of the final model by training all 500 samples. The network was evaluated by the participants in the test group (200 samples). The networks trained by ADC images are named as DL_ADC model, and the networks trained by T2-weighted images are named as DL_T2 model. The area under the curve (AUC) of receiver operating characteristic (ROC) were calculated. Two DL models were first trained by combining images from 1.5 Tesla and 3.0 Tesla scanners and then trained by images scanned at the 3.0 Tesla scanner only. The processed data and the weights of trained networks can be found at the following link (https://github.com/radiologypkucancer/rectal_MR_DL).

## Results

The 500 patients in the training group included 116 (23.2%) GRs and 384 (76.8%) non-GRs. The 200 patients in the test group included 60 (30%) GRs and 140 (70%) non-GRs. The distribution of GRs and non-GRs shows insignificant difference between the training group and the test group by Chi-square test (χ ^2^ = 3.510, P = 0.07) (P < 0.05 is considered significant difference). The clinical information was summarized in **Table 2**. The age, sex, pretreatment T-stage and N-stage show insignificant difference between GRs and non-GRs in both training group and test group. The pretreatment mean ADC value inside tumor ROI shows significant difference between GRs and non-GRs in both training group (T = 3.937, P = 0.000) and test group (T = 4.439, P = 0.000).

**Table 2:** Characteristics of participants in training group and test group.

Characteristics****	Training group****		Test group****	
	GRs (n = 116)	Non-GRs (n = 384)	GRs (n = 60)	Non-GRs (n = 140)
**Age (years)**	P = 0.868 (t = 0.167)		P = 0.519 (t = 0.646)	
	56.14 ± 11.76	55.94 ± 10.84	57.73 ± 10.80	56.68 ± 10.50
**Gender (%)**	P = 0.303 (χ ^2^ = 1.061)		P = 0.124 (χ ^2^ = 0.724)	
** Male**	71 (61.2)	255 (66.4)	37 (61.7)	90 (64.3)
** Female**	45(38.8)	129 (33.6)	23 (38.3)	50 (35.7)
**Pretreatment T-stage**	P = 0.059 (χ ^2^ = 7.445)		P = 1.093 (χ ^2^ = 0.779)	
** T2**	17 (14.7)	30 (7.8)	3 (5.0)	7 (5.0)
** T3**	82 (70.7)	294(76.6)	44 (73.3)	93 (66.4)
** T4a**	5 (4.3)	31 (8.1)	8 (13.3)	26 (18.6)
** T4b**	12 (10.3)	29 (7.6)	5 (8.3)	14 (10.0)
**Pretreatment N-stage**	P = 0.406 (χ ^2^ = 3.999)		P = 0.688 (χ ^2^ = 2.260)	
** N0**	8 (6.9)	19 (4.9)	1 (1.7)	3 (2.1)
** N1a**	9 (7.8)	16 (4.2)	2 (3.3)	5 (3.6)
** N1b**	18(15.5)	51 (13.3)	7 (11.7)	8 (5.7)
** N2a**	27 (23.3)	103 (26.8)	21 (35.0)	49 (35.0)
** N2b**	54 (46.6)	195 (50.8)	29 (48.3)	75 (53.6)
**ADC (10^-3^mm^2^/sec)**	P = 0.000 (T = 3.937)		P = 0.000 (T = 4.439)	
	1.05 ± 0.17	1.11 ± 0.13	1.01 ± 0.16	1.11 ± 0.10

After cross-validation, the optimal hyperparameters were determined as follow: the learning rate is 3 × 10^-5^, the decay rate is 1 × 10^-4^, and the batch size is 30 for both DL_ADC and DL_T2 models. The mean AUC of DL_ADC model reached maximum after training 2 000 epochs and then declined. The mean AUC of DL_T2 model reached maximum after training 1,200 epochs and then declined. Finally, the models were trained by the whole training group with the optimal hyperparameters above and evaluated by the participants in the test group.

**Figure 4:**
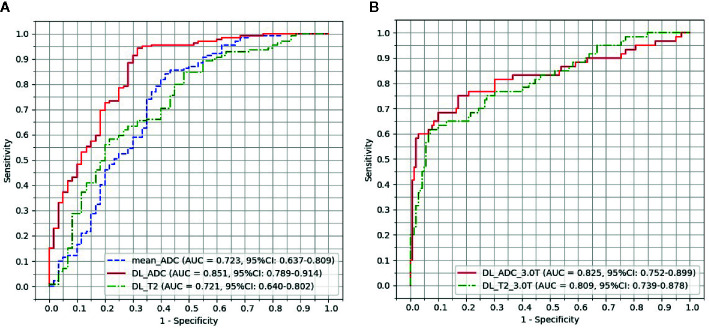
Receiver operating characteristic (ROC) curve analysis for the prediction of good responders to neoadjuvant chemoradiotherapy. **(A)** Models trained by the combination of images acquired from both 1.5 Tesla and 3.0 Tesla scanners. **(B)** Models trained by the images acquired from a 3.0 Tesla scanner only.

ROC curves of test group were plotted in **Figure 4** by comparing the pathological ground truth with the mean ADC value, GR probability predicted by DL_ADC model and DL_T2 model in the test group. The cut-off value was determined by maximizing the Youden’s index (sensitivity+specificity-1). **Figure 4A** shows the models trained by the combination of images from 1.5 Tesla and 3.0 Tesla. The AUC of mean ADC value is 0.723 (95% CI: 0.637–0.809). The cut-off value of mean ADC is 1.07 × 10^-3^ mm^2^/sec. The AUC of DL_ADC is 0.851 (95% CI: 0.789–0.914), significantly larger (P = 0.018, Z = 2.367) than the AUC of mean ADC value by DeLong test (23). The AUC of DL_T2 is 0.721(95% CI: 0.640–0.802), significantly lower than DL_ADC model (P = 0.000, Z = 3.554). **Figure 4B** shows the models trained by the images from a 3.0T scanner only. The AUC of DL_ADC_3.0T is 0.825 (95% CI: 0.752–0.899), and the AUC of DL_T2_3.0T is 0.809(95% CI: 0.739–0.878). There is no significant difference between two models (P = 0.676, Z = 0.418). Sensitivity, specificity, positive predictive value (PPV) and negative predictive value (NPV) were summarized in **Table 3**.

**Table 3:** Performance of therapy response prediction in the test group by mean ADC and four different deep learning (DL) models.

Model****	AUC****	Sensitivity %****	Specificity %****	PPV %****	NPV %****
**mean ADC**	0.723(0.637–0.809)	84.3(77.2–89.9)	58.3(44.9–70.9)	82.5(75.3–88.4)	61.4(47.6–74.0)
**DL_ADC**	0.851 (0.789–0.914)	94.3(89.1–97.5)	68.3 (55.0–79.7)	87.4(81.0–92.3)	83.7(70.3–92.7)
**DL_T2**	0.721(0.640 – 0.802)	92.9(87.3–96.5)	36.7(24.6–50.1)	77.4(70.3–83.5)	68.8(50.0–83.9)
**DL_ADC_3.0 T**	0.825(0.752–0.899)	89.3(82.9–93.9)	68.3(55.0–79.7)	86.8(80.2–91.9)	73.2(59.7–84.2)
**DL_T2_3.0 T**	0.809(0.739–0.878)	92.9(87.3–96.5)	61.7(48.2 – 73.9)	85.0(78.3–90.2)	78.7(64.3–89.3)

AUC, area under the curve; PPV, positive predictive value; NPV, negative predictive value.

## Discussion

In this study, we proposed a deep learning method to predict the response to NCRT by only using the pretreatment MRI data. Compared with the strategy that uses both pretreatment and posttreatment data, the method may predict the response to NCRT before the initiation of treatment. It enables a chance to modify the treatment plan as early as possible. NCRT in this study generally takes 30 days, which is a crucial period during the treatment. A patient predicted as non-GR may require intensified chemoradiotherapy or combination with other treatments to avoid tumor progression during therapy and increase the chance of organ preservation. If the prediction accuracy could be further improved, individualized strategy could be used to treat LARC to optimize the benefit of each patient.

It has been debated whether pretreatment ADC image can predict GRs. Dzik-Jurasz et al. have reported that a GRs group has significantly lower pretreatment ADC than non-GRs (24). Sun et al. have also found that pretreatment ADC is significantly lower in the LARC patients with histopathologic downstaging than no downstaging (25). But DeVries et al. have found that pretreatment ADC values in GRs are almost identical to non-GRs (26). Bulens et al. have claimed that there is no significant difference in pretreatment ADC between GRs and non-GRs (11). In this work, the mean ADC value shows significant difference between GRs and non-GRs in both training group and test group. But the prediction of GRs by mean ADC value is unsatisfactory (AUC = 0.723). The cut-off value of mean ADC in this work is slightly smaller than that in reference (24, 25), but much larger than that in reference (26). The variance in measured ADC values may come from ROI delineation, which is probably smaller or larger than the true tumor region. In this work, most of the uncertain pixels on the boundary between normal tissues and tumor were excluded from ROI during delineation, resulting in the decrease in ADC measurement. DL model produces a significantly larger AUC of 0.851 than mean ADC value. It suggests that a lot of information hidden in the ADC image is useful for the prediction of GRs. The result of DL model is also better than the reported results predicted by radiomics methods (19, 21). The advantage of DL method is its ability to adaptively extract features according to the data instead of using predefined features. Different from the Fu’s work (AUC = 0.73, n = 43) that uses VGG network (22) pretrained by natural images, the network in this work was trained by real images of rectal cancer. Therefore, the networks are more likely to capture some effective features for GR/non-GR differentiation.

In this study, an architecture of networks was designed in this study by paralleling a max-pooling layer with a center-cropping layer to extract features from different scales. The max-pooling layer downsamples the image into its 1/4 size and the center-cropping layer gets the central 1/4 part of the image. Several approaches have been proposed to handle differently scaled data, such as pyramid feature extraction or dilated convolution (27, 28). The advantage of these networks is the segmentation or detection of multiple objects with different sizes. Since manual delineation is used in this study, our task focused on classification rather than segmentation or detection. In addition, the region of rectal tumor is the only target in the image and located in the central area of the image patch after zero-padding. Therefore, a center-cropping layer was designed in this study to reduce the complexity of the networks.

In this work, DL model was trained by images scanned at both 1.5 Tesla and 3.0 Tesla scanners. Unlike T1-weighted, T2-weighted or diffusion-weighted images that depend on magnetic field strength and scanning parameters, ADC values are the inherent characteristics of the tissues that are generally unchanged in different scanning conditions. Therefore, ADC images from different scanners in this work could be directly inputted into the network without normalization. When T2-weighted images were used, normalization is indispensable. Because MRI signal is nonlinearly related with proton density, relaxation time such as T1 and T2, scanning parameters such as time of repetition (TR) and time of echo (TE), it is a challenging problem to normalize MRI image scanned at different magnetic field strength or parameters. In this study, T2-weighted images were normalized to the range between 0 and 1. However, results show that the AUC of DL_T2 model is significantly lower than DL_ADC model. After inclusion of T2-weighted images acquired at 1.5 Tesla scanner, the AUC is lower than the model trained by images from the 3.0 Tesla scanner only. It suggests that the normalization method is inadequate to eliminate the difference of MRI data acquired at different scanning conditions. Signal inconstancy is still an unsolved problem in training DL network if images from multiple sources are used. On the contrary, the AUC of ADC model slightly increased after combining images from two scanners. It is probably due to the increase of the training samples for deep learning. The results suggest that ADC images are less affected by the variance among different scanners. ADC images could be good candidates for the construction of deep learning models by the data from multiple sources.

Retrospective study is the main limitation of this work. A respective study with external validation cohort at multiple centers may further demonstrate the performance of the model in clinical practice. Data augmentation was used in this work to increase the size of training samples. Although augmentation is a common trick for DL, real samples should be used if more data are available. Another limitation of this work is manual delineation that is both subjective and time-consuming. Automatic segmentation is a promising solution, but the accuracy and stability still require improving.

## Conclusion

DL model based on pretreatment ADC images is potentially useful in the prediction of response to NCRT. The method could be used to individualize the treatment plan for LARC patients before the start of NCRT.

## Data Availability Statement

The raw data and codes supporting the conclusions of this article are available at https://github.com/radiologypkucancer/rectal_MR_DL.

## Ethics Statement

The studies involving human participants were reviewed and approved by Ethics Committee, Peking University Cancer Hospital and Institute. Written informed consent for participation was not required for this study in accordance with the national legislation and the institutional requirements.

## Author Contributions

H-TZ, X-YZ, and Y-SS: study conception and design. X-YZ and Y-JS: data collection and analysis. H-TZ: image processing and modeling. H-TZ and X-YZ: manuscript writing. X-TL: statistical analysis. All authors contributed to the article and approved the submitted version. H-TZ and X-YZ contribute equally to the manuscript.

## Funding

This study was supported by National Natural Science Foundation of China (81971584, 81501464, 91959116), Beijing Municipal Administration of Hospitals Clinical Medicine Development of Special Funding Support (No. ZYLX201803), ‘Beijing Hospitals Authority’ Ascent Plan, (Code:20191103), National Key R&D Program of China (2019YFC0117705, 2017YFC1309101, 2017YFC1309104), Capital's Funds for Health Improvement and Research (2020-1-2151), Beijing excellent talents training project (2018000021469G260).

## Conflict of Interest

The authors declare that the research was conducted in the absence of any commercial or financial relationships that could be construed as a potential conflict of interest.
